# Supplementary figures and images for: Deconvolute individual genomes from metagenome sequences through short read clustering
Source: PeerJ. 2020 Apr 8;8:e8966. doi: 10.7717/peerj.8966 (PMC7150542; doi:10.7717/peerj.8966)

Memory saved in Mock and CAMI dataset

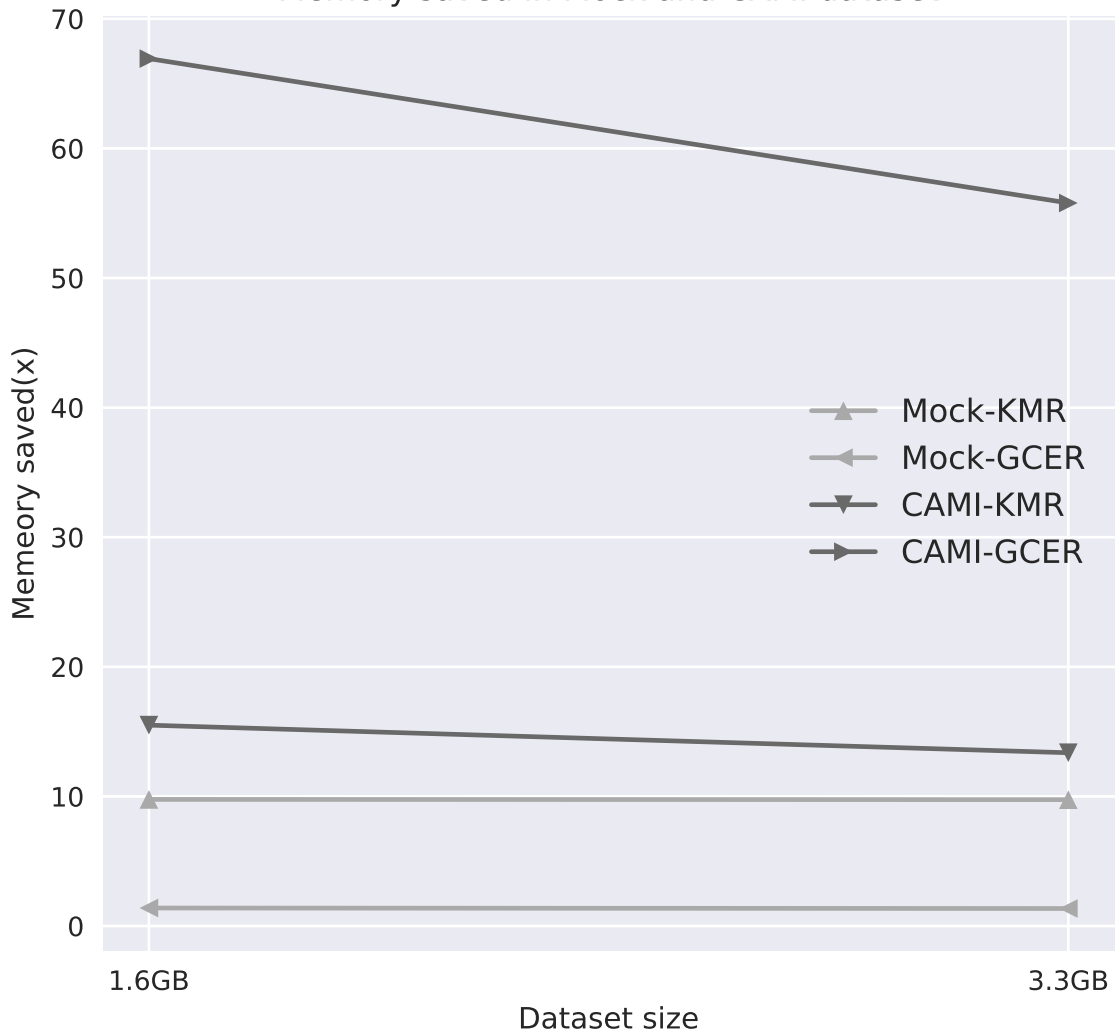

Time saved in Mock and CAMI dataset

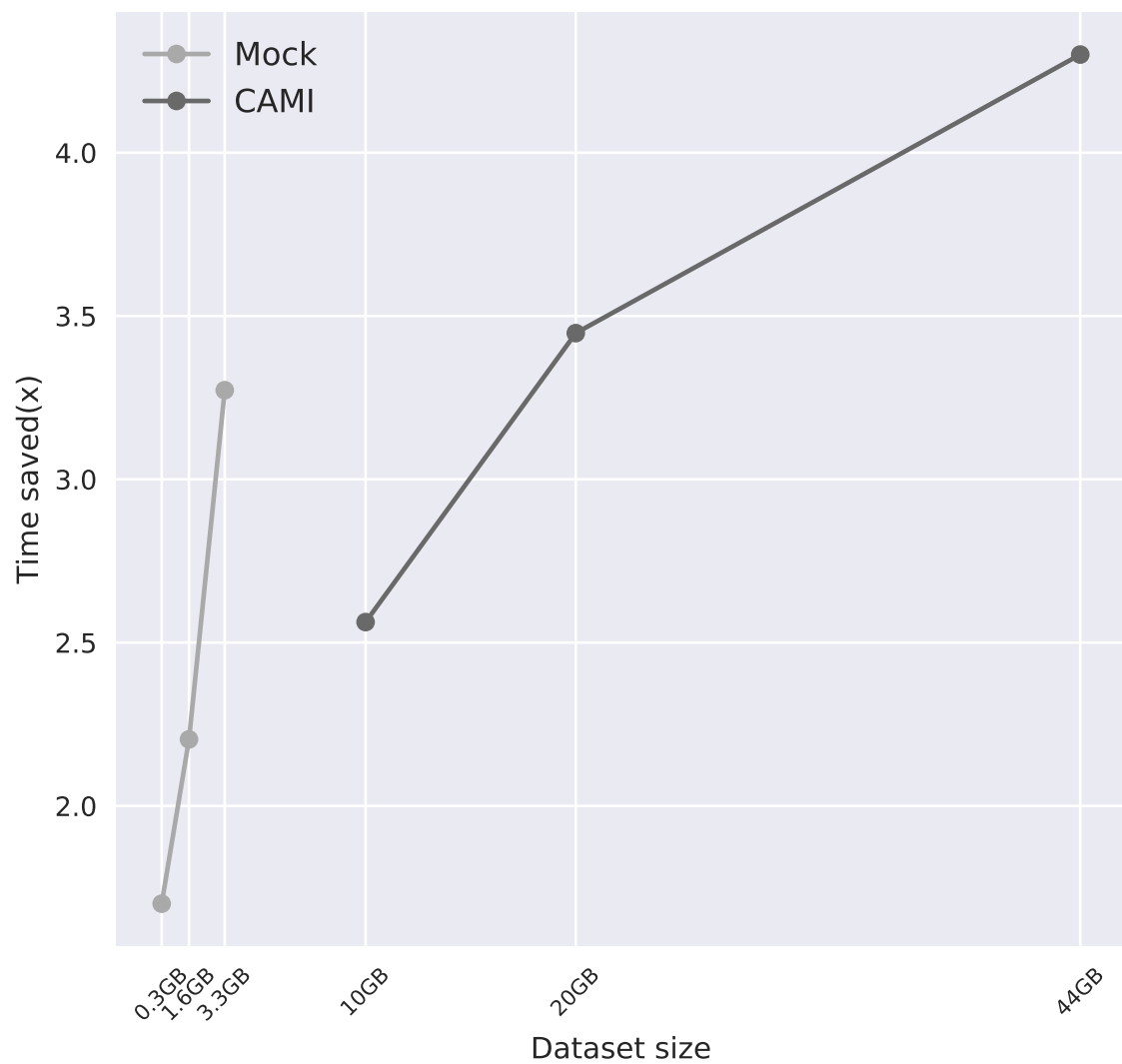

Supplement: Supplemental Information 1 — Left: Memory reduction (y-axis, in folds) with minimizers relative to default (k-mers) during the KMR and GCER steps, on both Mock and CAMI2 datasets. Two dataset sizes were shown (x-axis). Right: Overall speed up (y-axis, in folds) with minimizers relative to default (k-mers). The size of data is shown on x-axis. [file peerj-08-8966-s001.pdf]
